# UV light assisted antibiotics for eradication of *in vitro* biofilms

**DOI:** 10.1038/s41598-018-34340-8

**Published:** 2018-11-05

**Authors:** Aikaterini Argyraki, Merete Markvart, Camilla Stavnsbjerg, Kasper Nørskov Kragh, Yiyu Ou, Lars Bjørndal, Thomas Bjarnsholt, Paul Michael Petersen

**Affiliations:** 1 Department of Photonics Engineering, Technical University of Denmark, Frederiksborgvej 399, DK-4000 Roskilde, Denmark; 2 Cariology and Endodontics, Department of Odontology, Faculty of Health and Medical Sciences, University of Copenhagen, Nørre Allé 20, DK-2200 Copenhagen N, Denmark; 3 Department of Immunology and Microbiology, Costerton Biofilm Center, Faculty of Health and Medical Sciences, University of Copenhagen, Blegdamsvej 3B, DK-2200 Copenhagen N, Denmark; 4 Department of Clinical Microbiology, Rigshospitalet, Juliane Maries vej 22, 2100 Copenhagen Ø, Denmark

## Abstract

The overuse of antibiotics is accelerating the bacterial resistance, and therefore there is a need to reduce the amount of antibiotics used for treatment. Here, we demonstrate *in vitro* that specific wavelengths in a narrow range around 296 nm are able to eradicate bacteria in the biofilm state (grown for 24 hours) more effectively, than antibiotics and the combination of irradiation and antibiotics is even better, introducing a novel concept *light assisted antibiotics*. The investigated wavelength range was 249 nm to 338 nm with an approximate step of 5 nm. The novel concept that consists of a UV irradiation treatment followed by a tobramycin treatment can significantly reduce the amount of antibiotics needed for eradicating mature bacterial biofilms. The efficiency of the proposed light assisted antibiotics method was compared to combinatory antibiotic treatment and highly concentrated antibiotic monotherapy. The eradication efficacies, on mature biofilms, achieved by light assisted antibiotic and by the antibiotic monotherapy at approximately 10-fold higher concentration, were equivalent. The present achievement could motivate the development of light assisted antibiotic treatments for treating infections.

## Introduction

There is a worldwide increasing awareness and concern about the antibiotic resistance, which in the future could prevent effective treatment of a large number of infectious diseases^1^. The emergency of antibiotic resistance has evoked a “turn” towards antibiotics control/reduction programs^2^. Moreover, biofilm infections are highly persistent to the immune response and known for their tolerance to antibiotic treatments^3^. Alternative approaches, different than conventional antibiotic treatments, are gaining increasing interest and light based treatments^4^ are unconventional strategies for biofilm eradication. Bak *et al*. has reported disinfection potential of catheter lumens by UVC LEDs^5^. Recently, we have observed that UVB irradiation is more efficient than UVC, in eradicating biofilms plated on cellulose nitrate membrane filters^6^. However, the ultraviolet (UV) wavelength dependent eradication efficiency for Pseudomonas aeruginosa biofilms has not been reported. Apart from UV light, also exposure to blue light has been proven to have antimicrobial effect, without the requirement of exogenous photosensitizers present^7^.

UV light emitting diodes (LEDs) are progressing as light source options in biomedical applications mainly due to their flexibility in spectral design and ease in operation. AlGaN LEDs are continuously progressing as UV light sources. A wavelength as short as 210 nm has been achieved with pure AlN LEDs^8^. External quantum efficiency (EQE) of UV LEDs is continuously improving as both internal quantum efficiency (IQE) and light extraction efficiency (LEE) are boosted by various techniques like: migration-enhanced metalorganic chemical vapor deposition^9,10^, ammonia pulsed-flow method^11,12^ and nanowires^13^, or surface plasmons^14^ and aluminum reflective electrodes^15^, respectively. EQE above 10% has been reported when approaches for improving IQE and LEE are applied in combination^16^.

Combinatory therapies seem to be the solution for combating tolerant biofilms present in chronic infections^17,18^. Particularly, if the combinatory treatments enact complementary mode of actions then synergy can be expected, and therefore, better eradication efficiency^19–21^.

Biofilms are complex formations created by bacteria to improve their chances of survival. More specifically, biofilms can be 10 to 1000 times more tolerant than the planktonic phenotype^22^. The organization and composition existing in a biofilm makes the treatment of biofilm infections challenging since bacteria in biofilms can employ specific mechanisms to tolerate bactericidal treatments. The origin of biofilm tolerance is mostly caused by low metabolic activity of the bacteria within the biofilm, but it also has a genetic basis^23,24^. Furthermore, the physical barrier of the biofilm matrix, limits the diffusion of molecules^25^ into the biofilm, and reduces antimicrobial penetration.

Common sites of biofilm infections in the human body are the oral cavity, e.g. caries is the most frequent disease affecting human health^26^, burdening billions of individuals with pain, limited masticatory functions and impaired aesthetics. In particular, the deep carious lesions^27^, as well as its sequelae, the infected root canal associated with biofilm infection, represent targets for improved antimicrobial strategies and represent unsolved demanding challenges within the dental community^28^. Also within, the urinary system, the lungs of cystic fibrosis patients and chronic wounds biofilm infections are common. Additionally, when medical devices, like catheters, endoscopes, tissue fillers, implants, iatrogenic placed endodontic root filling materials, etc. are inserted into the body, the risk for chronic biofilm infection increases^29^.

There is urgent need to enable elimination of chronic biofilm infections without utilizing excessive amounts of antibiotics. The present study could assist in developing light assisted combinatory treatments, consisting of irradiation in combination with antibiotics. The scope is to achieve total biofilm eradication and reduce the amount of antibiotics needed for treating infections.

In the present work we aimed to demonstrate the wavelength dependent survival of 24 h (h) grown *P*. *aeruginosa* biofilms in the range 249 nm to 338 nm with an approximate step of 5 nm. The photon rate was 0.0036 mol/m^2^; corresponding to a radiant exposure of 1.700–1.260 J/m^2^. We report remarkable eradication (eradication log higher than 8) for the 24 h old biofilms after irradiation in the 292–306 nm range for 0.0495 mol/m^2^ photon rate, corresponding to a radiant exposure 17.500–21.100 J/m^2^. A UVC treatment at that exposure level could have negative implications to the healthy tissue infected by the biofilm; therefore, the wavelengths tested at this higher level of radiant exposure were restricted only to the UVB and UVA region.

To demonstrate the antimicrobial effect of the irradiation method, we compared the irradiation strategy with two types of antibiotics that are well recognized for their usage against P. aeruginosa biofilm infections, namely tobramycin^30^ and colistin^31^ both as monotherapy. The hypothesis was no difference of the eradication efficacy of the three different treatments: UVB or antibiotic monotherapies, on *in vitro* P. aeruginosa biofilms either grown for 24 h or 48 h.

Light assisted antibiotic treatments for biofilm infections could be a method to improve the therapy of biofilm infections in the future, since light has been shown to have antibacterial action at several wavelengths^7^: UVC, UVB, UVA, blue, infrared. Here, we demonstrate the light assisted antibiotic principle in specific, by applying irradiation with a UV LED exhibiting central wavelength at 296 nm and by subsequently administrating topically tobramycin, to combat bacteria of P. aeruginosa biofilms grown for 24 h (immature) or 48 h (mature). The biofilm eradication of the light assisted tobramycin is compared to the effect achieved by 10-fold more concentrated tobramycin, as well as combinatory antibiotic treatments, consisting of tobramycin and colistin at two concentration levels. Tobramycin and colistin are known to be used in combination due to the increased effectiveness of the combined treatment compared to monotherapy^18^. The hypothesis was no difference of the eradication efficacy of the four different treatments: light assisted tobramycin or 10-fold more concentrated tobramycin monotherapy and combinatory antibiotics at two different concentration levels, on *in vitro* P. aeruginosa biofilms either grown for 24 h or 48 h.

The proposed method is relevant for combating biofilms and could assist in developing new combinatory therapies consisting of light application and usage of antibiotics to improve treatment of chronic biofilm infections in complex ecosystems e.g. the dental root canal system^32^, as well as treating postoperative infection adjacent to biomedical implants.

## Results

### Wavelength dependent survival of biofilms

**Figure 1:**
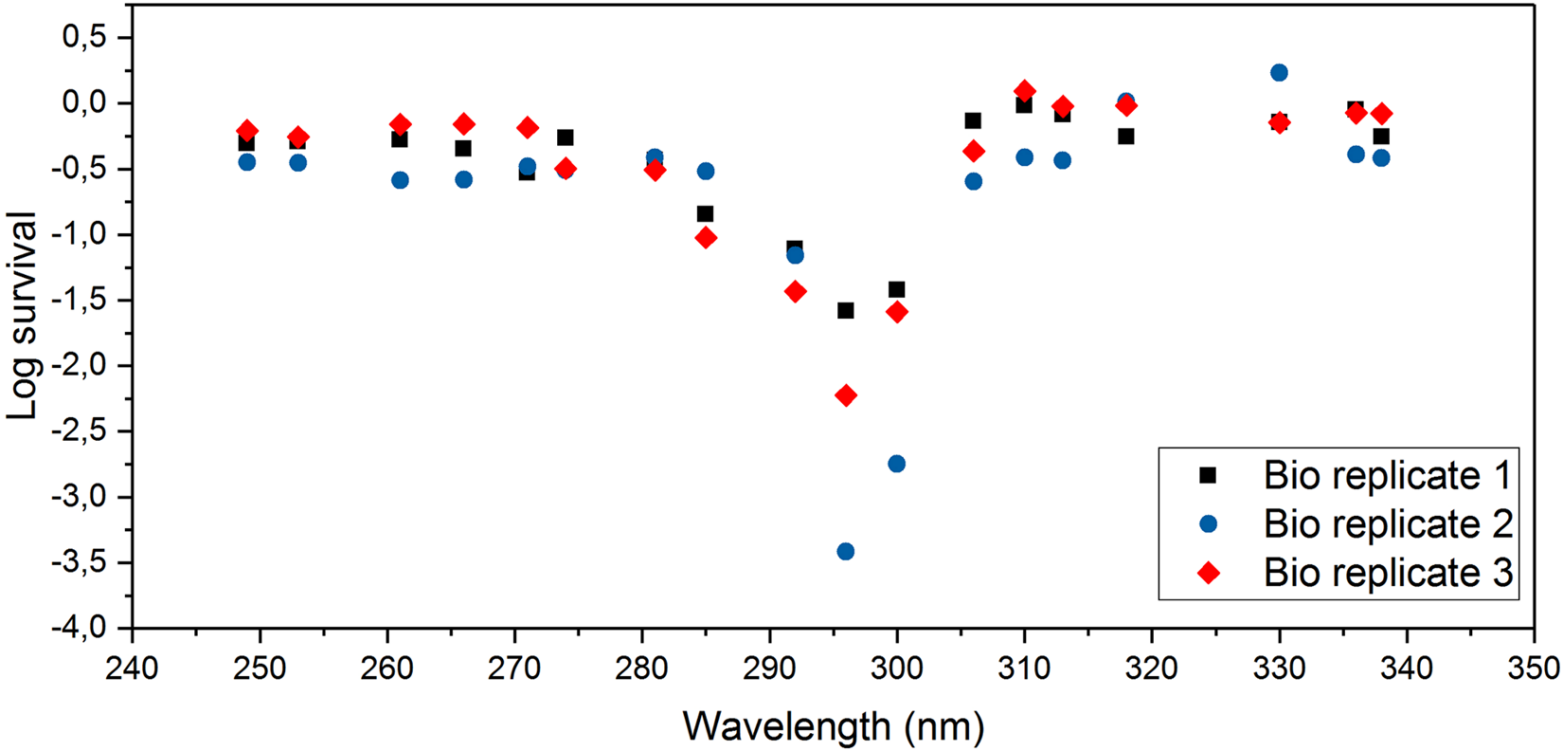
Wavelength dependent survival of *P*. *aeruginosa* biofilm grown for 24 h. The photon rate delivered on the biofilms by the UV LEDs was 0.0036 mol/m^2^, corresponding to a radiant exposure 1.260 J/m^2^ to 1.700 J/m^2^, respectively, for the wavelengths 338 nm to 249 nm. Three biological replicates were generated for all treatments.

The survival of the biofilms after each treatment, was calculated according to Eq. 1

1 $$\mathrm{log}\,survival=\,\mathrm{log}(\frac{{N}_{treated}}{{N}_{contol}})=-\,\mathrm{log}\,eradication$$

where Ntreated is the number of colony forming units (CFUs) per micropore filter after a treatment is applied to the biofilm, and N control is the number of CFUs per micropore filter of non-treated samples. The wavelength dependent survival of the *P*. *aeruginosa* biofilms grown for 24 h is presented in Fig. 1. All treatments were repeated on three different biological replicates.

UVA irradiations were performed with LEDs having central wavelengths from 318 nm to 338 nm, UVB irradiations from 281 nm to 313 nm, covering the whole UVB spectral range, and UVC irradiations from 249 nm to 274 nm. It was observed that the estimated CFUs of the UVA treated samples, independently of wavelength applied, were similar to non-treated samples (control). The log survival was 0.13 ± 0.17. Independently of wavelength, UVC treated samples exhibited a log eradication 0.36 ± 0.15 (Eq. 1). UVB irradiated samples to the contrary exhibited strong dependence of wavelength and eradication ability and less CFUs were observed, especially for the range 292 to 300 nm. The treatment with the diode having central wavelength at 296 nm exhibited the strongest eradication potential on *P*. *aeruginosa* 24 h grown biofilms with log eradication 2.39 ± 0.78.

**Figure 2:**
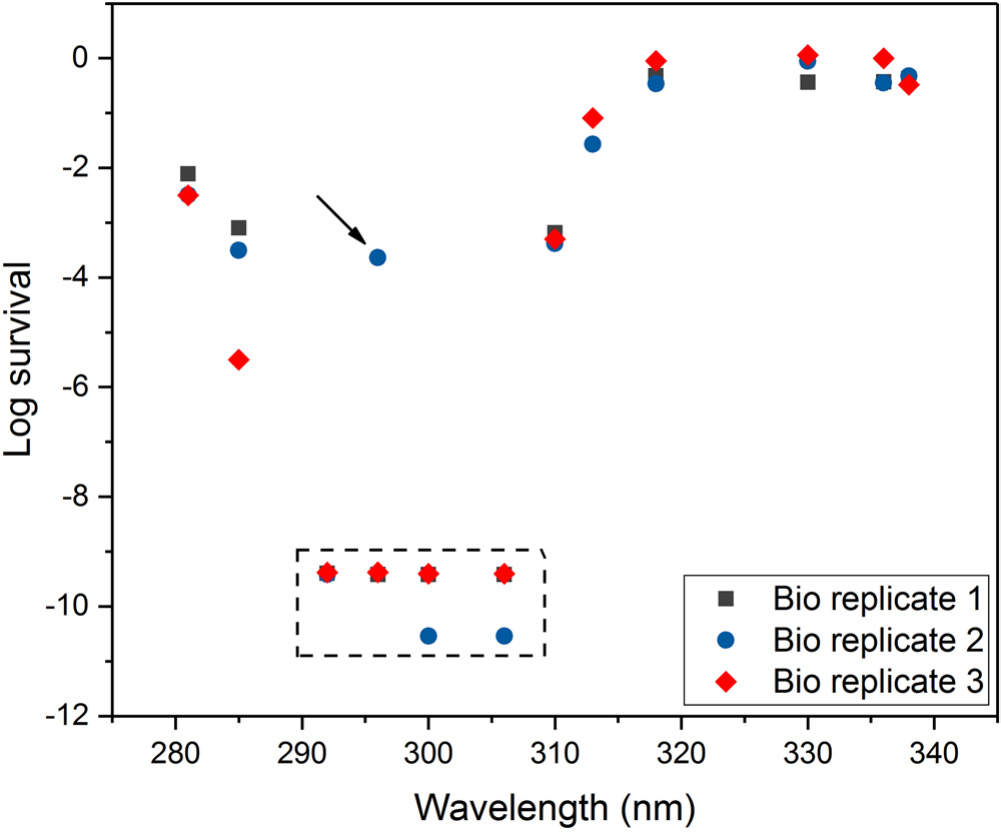
Survival of biofilm after UVB or UVA LED exposure, the level of exposure was equivalent to what can be received by sunlight. The photon rate delivered on the biofilms by the UVB and UVA LEDs was 0.0495 mol/m^2^, corresponding to a radiant exposure 17.500 J/m^2^ to 21.100 J/m^2^. The biofilms were *P*. *aeruginosa* and grown for 24 h. The peculiar observation indicated by the black arrow led us to perform several repetitions of this specific treatment; all repetitions resulted in log eradication higher than 8. The dashed box comprises measurements corresponding to zero counts. The measurements in the dashed box in Fig. 2 correspond to zero counts, i.e. complete eradication of the biofilm.

The eradication ability at a radiant UVB exposure, equivalent to 12 h of summer sunlight in Northern Europe, was remarkable (eradication log higher than 8) for the wavelength range 292–306 nm (Fig. 2). The observed result suggested that a UVB radiant exposure of that level and at this wavelength range could also enact eradication effects on mature biofilms that are known for their increased tolerance to antibacterial treatments.

### UVB irradiation treatment versus topically administrated antibiotics

Three treatments were applied on *P*. *aeruginosa* biofilms grown for 24 h or 48 h, namely UVB irradiation (sunlight equivalent, 20.000 J/m^2^), or topical administration of antibiotics; either tobramycin or colistin at one hundred times the minimal inhibitory concentration (MIC). Two-way analysis of variance (ANOVA) revealed that there was a significant difference in eradication of biofilms for the three treatments for 24 h grown biofilms. UVB, compared to colistin or tobramycin, was significantly better at eradicating 24 h immature biofilms with p. values of P < 0.0001 and P < 0.0001, respectively. No significant difference was observed for the 48 h grown biofilm.

**Figure 3:**
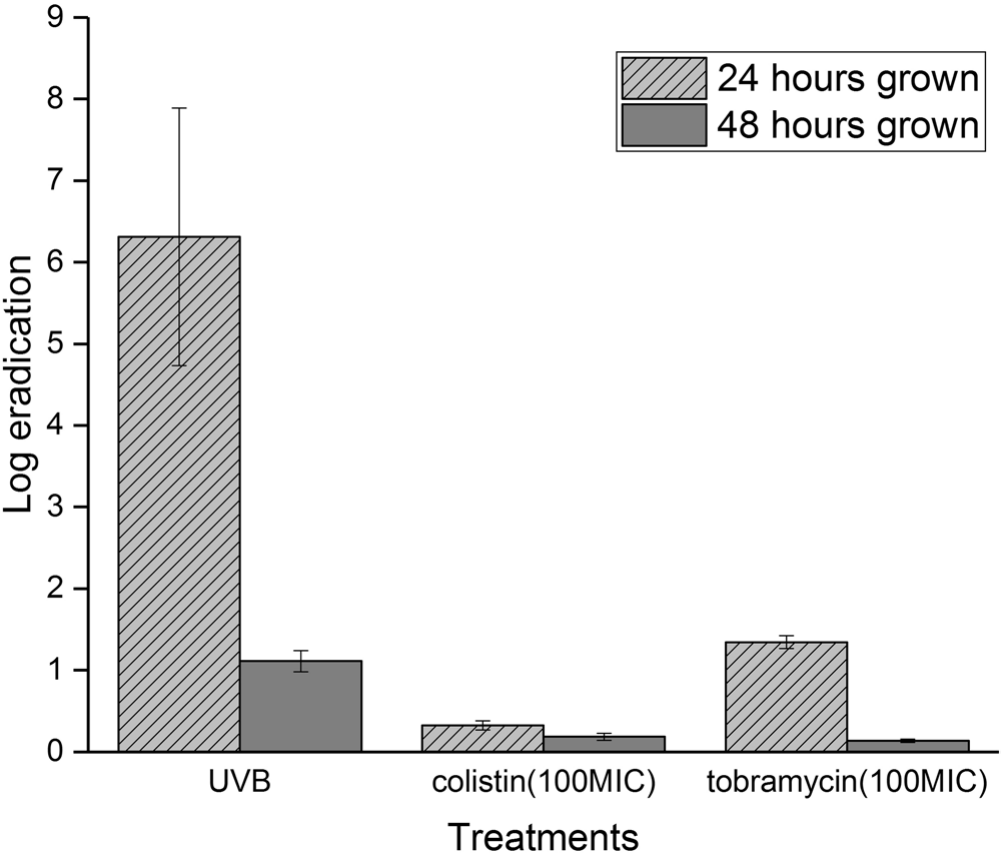
Eradication of biofilms after UVB irradiation treatment (sunlight equivalent), or topical administration of antibiotics; colistin or tobramycin at hundred times the MIC. The biofilms were either left to grow for 24 h before treatment or for 48 h. The error bars indicate the standard deviation as acquired by three biological replicates. “MIC” stands for minimal inhibitory concentration.

The biofilm eradication achieved by the three different treatments is presented in Fig. 3. The eradication achieved by the UVB irradiation treatment was lower on mature biofilms (1.11 ± 0.13 log eradication) than on immature biofilms (6.31 ± 1.58 log eradication). However, the UVB treatment was more efficient in eradicating biofilms than the antibiotics. The colistin treatment resulted in negligible eradication, independently of the biofilm growth. The tobramycin treatment enacted a moderate eradication on immature biofilms (1.34 ± 0.08 log eradication), but only negligible eradication on mature biofilms.

### Light assisted antibiotics

The CFU/filter values of the different treatments on 24 h and 48 h grown biofilms are presented in Table 1 together with the ones of untreated reference samples. The measurements include UVB, colistin (100MIC), tobramycin (100MIC), tobramycin (1000MIC), tobramycin (100MIC) + colistin (100MIC), tobramycin (1000MIC) + colistin (300MIC), UVB + tobramycin (100MIC). All treatments were repeated on three different biological replicates. For each biological replica, two technical replicas were applied and the average CFU/filter value of the two replicas was taken and shown in Table 1.

**Table 1:** CFU/filter values of *P*. *aeruginosa* biofilms after different treatments and the ones of untreated reference samples. *P*. *aeruginosa* biofilms grown by 24 h and 48 h were investigated. ‘24 hours’ and ‘48 hours’ refer to the biofilm state; ‘1’, ‘2’, ‘3’ refer to the biological replicates; ‘treat’ refers to the CFU/filter value after irradiations; and ‘ref’ refers to the CFU/filter value without treatment, as reference sample.

Treatment	Biofilms											
	1treat		1ref		2treat		2ref		3treat		3ref	
	24 hours	48 hours	24 hours	48 hours	24 hours	48 hours	24 hours	48 hours	24 hours	48 hours	24 hours	48 hours
UVB	1,67E + 02	2,59E + 08	1,85E + 09	4,25E + 09	8,31E + 04	3,14E + 08	2,64E + 09	4,47E + 09	1,50E + 02	4,87E + 08	3,65E + 09	4,49E + 09
colistin(100MIC)	1,16E + 09	2,73E + 09	2,14E + 09	4,63E + 09	1,97E + 09	3,41E + 09	4,29E + 09	4,75E + 09	1,73E + 09	3,18E + 09	4,07E + 09	4,85E + 09
tobramycin(100MIC)	1,08E + 08	3,40E + 09	2,14E + 09	4,63E + 09	1,57E + 08	3,32E + 09	4,29E + 09	4,75E + 09	2,03E + 08	3,69E + 09	4,07E + 09	4,85E + 09
tobramycin(1000MIC)	1,15E + 07	6,21E + 06	8,33E + 09	8,64E + 09	6,50E + 06	1,53E + 07	4,82E + 09	1,05E + 10	8,00E + 06	1,15E + 07	6,97E + 09	8,18E + 09
tobramycin(100MIC) + colistin(100MIC)	3,09E + 06	1,58E + 09	2,14E + 09	4,63E + 09	9,31E + 06	1,09E + 09	4,29E + 09	4,75E + 09	2,84E + 06	1,27E + 09	4,07E + 09	4,85E + 09
tobramycin(1000MIC) + colistin(300MIC)	0,00E + 00	3,94E + 05	8,33E + 09	8,64E + 09	0,00E + 00	3,05E + 05	4,82E + 09	1,05E + 10	0,00E + 00	3,24E + 05	6,97E + 09	8,18E + 09
UVB + tobramycin(100MIC)	0,00E + 00	1,35E + 07	1,85E + 09	4,25E + 09	0,00E + 00	2,36E + 07	2,64E + 09	4,47E + 09	2,70E + 03	1,96E + 06	3,65E + 09	4,49E + 09

**Figure 4:**
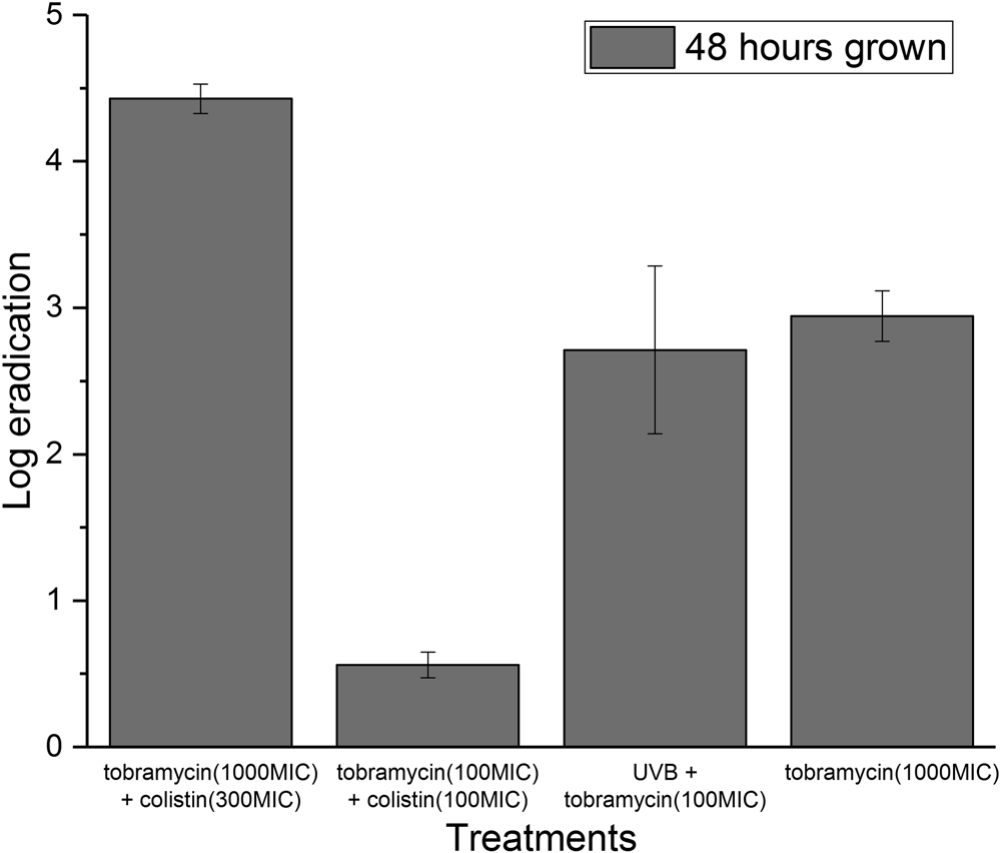
Eradication of biofilms achieved by light assisted tobramycin versus topical administration of 10-fold more concentrated tobramycin or combinatory antibiotics at two concentration levels. The biofilms were grown for 48 h before treatment. The error bars indicate the standard deviation as acquired by three biological replicates. “MIC” stands for minimal inhibitory concentration.

The biofilm eradication achieved by the suggested method of light assisted antibiotics on mature samples is presented in Fig. 4. The eradication achieved by combinatory administration of antibiotics, and after administration of highly concentrated monotherapy is also shown in Fig. 4. The one-way ANOVA analysis showed that there was a significant difference (p-value 0.0003) in the eradication achieved by the light-assisted treatment UVB + tobramycin (100MIC) shown in Fig. 4 and the treatment tobramycin (100MIC) shown in Fig. 3 for 48 h grown biofilms.

The light assisted tobramycin was even more effective than the combinatory antibiotics tobramycin and colistin (low-level concentration); and for 48 h biofilms the method of light assisted antibiotics was approximately equally as effective as 10 times higher concentration of tobramycin. Interestingly, the light assisted tobramycin treatment approached the eradication values achieved by topical administration of combinatory antibiotics at high concentrations.

## Discussion

*In vivo* and *in vitro* experiments have previously validated that the tolerance for biofilms are usually considerably higher (approx. 10–1000 times) than the planktonic bacterial cells^33^. Therefore, eradication of biofilms by conventional antibiotic administration can be challenging due to potential side effects or accumulated toxicity^34^. The demand for discovering alternative methods to eradicate biofilms, which may be involved in chronic infections, has been identified for a long time. Moreover, the need to develop treatments that would increase the vulnerability of biofilms to well-recognized therapeutic methods (e.g. antibiotics) has been acknowledged^35^. The established root canal infection associated with an apical periodontitis (i.e. inflammation surrounding the apical portion of the dental root) provides an area where light assisted antibiotics is applicable, as the prevailing combination of instrumentation and use of medicaments (e.g. sodium hypochlorite) do not completely sterilize the root canal system^36^. The concept of irrigation with MTAD (antibiotic solution) might be improved in combination with UV irradiation^37^. In addition, postoperative infections adjacent to biomedical dental implants remain a significant problem, which can lead to early implant failure^38^. The use of systemic antibiotics does not exert a significant preventive effect against these postoperative infections^39^. Thus, a final definition is still lacking of which drugs and administration regimens are the most effective antibiotic treatment protocol. The concept presented in this paper represents an option that could be used for treating established implant infections.

Several bacterial species have previously been studied for their sensitivity towards UV irradiation, like *Escherichia coli*^40^, and the optimal germicidal wavelength was found to lie within the UVC range with a maximum around 270 nm. However, the germicidal efficiency was studied for bacteria in the planktonic state. Bacteria in the planktonic state exist as individuals, while biofilms are aggregated bacteria. The peak of absorption of bacterial genetic material is located a few nanometers lower around 260–265 nm^41^, and this supported the hypothesis that direct UVC absorption by bacterial genetic material, inhibits normal replication, and results in bacterial eradication^42–45^.

In the present study it was shown that the eradication efficiency of UV irradiation on 24 h grown *P*. *aeruginosa* biofilms is wavelength dependent, and that the optimum region is located in the UVB range around 296 nm (292–300 nm). The maximum optical thickness for the 24 h grown *P*. *aeruginosa* biofilms treated in the present work was 75 ± 17 µm and for the 48 h grown 104 ± 12 µm. The maximum physical thickness was respectively 100 ± 23 µm and 138 ± 16 µm for the 24 and 48 h grown biofilms.

In the biofilm state, the smaller penetration achieved by shorter wavelengths is expected to reduce the eradication efficacy of UVC^6^. In the UVA region and longer wavelengths bacterial eradication is dictated only by indirect pathways like generation of reactive oxygen species, and therefore, the eradication efficiency is much lower^46^. UVB is located spectrally between the UVC and UVA regions; and involves elements from both indirect and direct bacterial impairment^47,48^. Studies on UVB lethality and mutagenesis of bacterial suspensions have shown that lethality occurs at a few nanometers longer wavelengths than mutagenesis^49^. Photons with UVB wavelengths in the 292–307 nm interval are expected to bring enough energy to break bonds like C-H and N-H^50^, essential for the tertiary structure of proteins and DNA^51^. In the human skin, free radical generation exhibits high efficiency for wavelengths around 303 nm (UVB range) and 355 nm (UVA range)^52^. Recently, a product with strong absorbance at 297 nm was reported by Puri *et al*.^53^ as present in *Methylobacter tundripaludum* supernatants in a quorum sensing dependent manner. The collected product was reported to have no distinguishable growth inhibitory activity against *E*. *coli* MG 1655 or *Bacillus subtilis* PY79, however, a possible growth inhibitory action of the product was not excluded for other bacterial species.

The route of antibiotic administration in the present study was that antibiotics were added directly to the biofilm. Therefore, the biofilm should be directly accessible to the antibiotic administration. The level of biofilm tolerance towards UV radiation or antibiotics may depend on how the biofilm has been cultured and which model was used. In the present work, it was demonstrated that when tobramycin at a concentration, which only imposed negligible eradication effect on 48 h grown biofilms, was administrated after UVB irradiation; it caused much larger eradication efficacy (2.71 ± 0.57 log eradication) and reached the same eradication values as 10-fold more concentrated tobramycin. The eradication effect from UVB alone on 48 h grown biofilms was significantly lower (1.11 ± 0.13 log eradication). This indicates a synergetic effect of light and antibiotics of which the exact mechanism remains to be understood and optimized according to the taxonomic diversity of the biofilm to be eradicated. The improved synergy for 48 h grown biofilms is interesting since the 48 h grown biofilm have fully developed antibiotic tolerance. It seems that the method of light assisted antibiotics is very suited for eradication of bacteria in mature biofilms with a fully developed tolerance.

## Conclusion

In conclusion, we have tested the efficiency of UV irradiation treatments to eradicate *P*. *aeruginosa* biofilms grown for 24 h in the wavelength range 249 nm to 338 nm with an approximate step of 5 nm. It was shown that the log survival of the biofilm was remarkably reduced for the wavelength range 292–306 nm, and the optimum was located at 296 nm. Moreover, we demonstrated that the UVB irradiation was more efficient than topical administration of antibiotics (colistin or tobramycin at 100 MIC) for eradicating biofilms grown for 24 h or 48 h.

A novel method was introduced, light assisted antibiotics, for eradicating mature biofilms and successfully reducing the amount of antibiotics used for disinfection. A specific light assisted antibiotic example was presented; namely, irradiation with a UV LED exhibiting central wavelength at 296 nm combined with topical administration of tobramycin at 100 MIC. This treatment reduced the bacterial load on 48 h grown biofilms by approximately 3 logs, equivalent to the effect as that achieved by administrating 10-fold more concentrated tobramycin (1000 MIC). The eradication achieved by the treatment was observed to be more effective than combinatory antibiotic treatment, 100 MIC of colistin plus 100 MIC of tobramycin. The present study can assist in developing new combinatory treatments consisting of light and usage of antibiotics to improve treatments of chronic biofilm infections within chronic wounds or within the infected root canal system treating infections in the jaw.

## Methods

### Biofilm preparation

The bacterial strain used in the experiments was *P*. *aeruginosa* PAO1 obtained from the Pseudomonas Genetic Stock Center (strain PAO0001, www.pseudomonas.med.ecu.edu)^54^. The micropore assay methodology used to form the biofilms was based on Bjarnsholt *et al*.^55^, in brief: The Micro-pore assay is based on biofilms growing on a micropore filter on AB minimal medium supplemented with glucose and mixed with 2.0% agar (AGBT) (Substrate Department at the Panum Institute, Denmark). The Cellulose nitrate membrane filters with pore size 0.2μm and diameter 25 mm purchased from Whatman GmbH (Germany) were placed on top of the ABGT plate. Bacteria from overnight cultures are propagated on the micro-pore filters as spots of 20 µl bacterial suspensions and incubated at 37 °C. For a mature biofilm to develop the filters were transferred to a fresh AB minimal agar plate after 24 h. Treatments were applied to the biofilms after either 24 h (immature biofilm) or 48 h (mature biofilm) incubation in total, at 37 °C^56^. It is observed that the control biofilms have a bacterial density in a level of 10^9^–10^10^ CFU/filter.

### Biofilm thickness measurement

Maximum biofilm thickness was measured by staining filter biofilms with 10 µl (2.5 μM) of universal fluorescent nucleotide stain Syto9 (Invitrogen, USA). Biofilms were imaged as a line-box, measuring 10171 µm (X) × 60.25 µm (Y) x 294 µm (Z) with 5 µm increments in the Z direction on confocal microscope (Zeiss Imager.Z2 microscope with LSM 710 CLSM running Zeiss Zen 2010 v. 6.0. (Zeiss, Germany)). A 488 nm laser was used for excitation and a 505–525 nm filter with a peak a 509 nm for emission. These settings provided a profile image as a cross-section from edge to edge of the filter biofilm. With the use of Imaris 9.0 (Bitplane, Schweiz) thickness of the biofilm was measured. In the format “section view” the profile of the biofilm could be measured as the length from the top point to the base of on the filter membranes surface. The biofilms were measured at the highest point at the edge, approx. 300 µm from edge on each side of the biofilm; additionally, the center of the biofilm was measured as well.

### UV Irradiations

**Figure 5:**
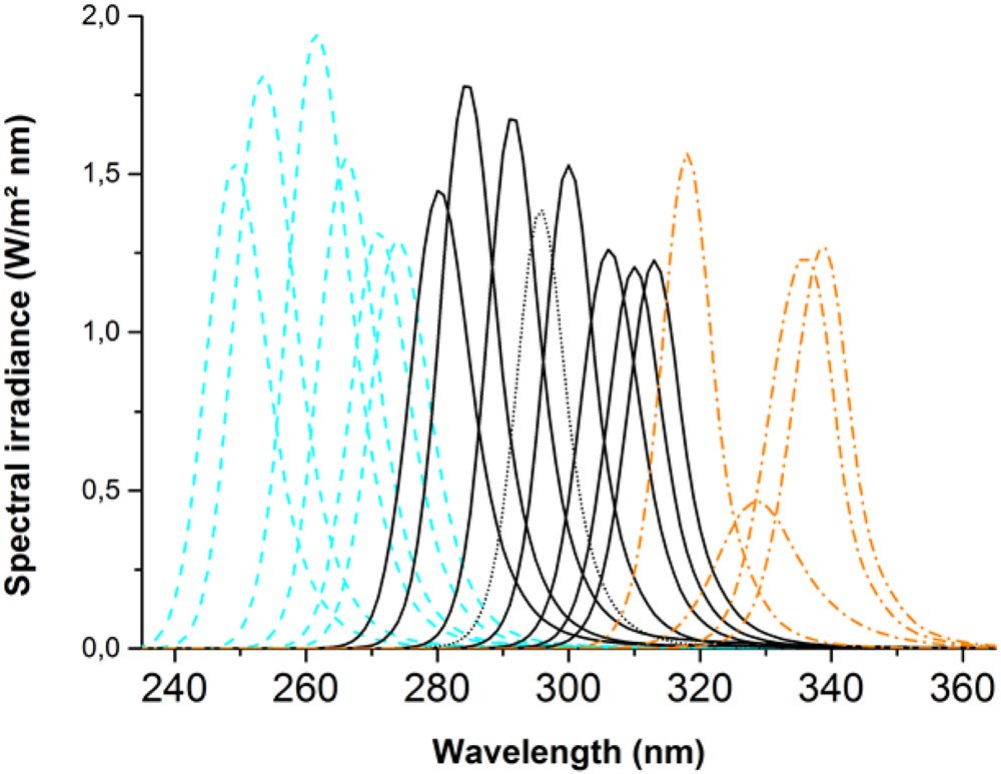
Spectral irradiance of UV LEDs used for determination of the optimal biofilm eradication wavelength. The UVC diodes are indicated with cyan color and dash line. The UVB diodes are indicated by black line, and black short dash for the optimal diode, central wavelength at 296 nm. UVA diodes are indicated by orange dash dot line.

The UV LEDs that were used to perform the irradiation treatments were purchased from Sensor Electronic Technology, Inc (SETi, Columbia, SC, USA). The spectral irradiance of the diodes used to determine the optimal biofilm eradication wavelength, is depicted in Fig. 5. The spectral irradiance was measured by an External Optical probe (EOP-146, Instrument Systems GmbH, Munich, Germany) and a monochromator. The spectrometer used was a SPECTRO 320 (D) Release 5 (Instrument Systems GmbH). The exact protocol for measuring the spectral irradiance can be found in Barnkob *et al*.^57^. The irradiance delivered on the biofilms was measured with a portable radiometer (NIST Certified UV Radiometer) at a distance (1.5 ± 0.1 cm). The distance between the biofilms and the UV LEDs was 1.5 ± 0.2 cm for all exposures; the error originates from the agar height, on which the filter carrying the biofilm was placed. The biofilms were kept in a UV free environment, when not treated. The UV treatment was conducted at a temperature of 20 °C for all the samples.

### Antibiotic treatments

The antibiotics used in this study were tobramycin (Eurocept International, Netherlands) and colistin sulfate salt (Sigma-Aldrich, USA). The MIC concentration for colistin was determined as 0.8 µg/mL and for tobramycin 1 µg/mL. The antibiotic treatments delivered were: 100 µg/mL tobramycin (100 MIC); 80 µg/mL colistin sulfate salt (100 MIC); tobramycin and colistin high (tobramycin + colistin high): 1000 µg/mL tobramycin (1000 MIC) and 250 µg/mL colistin sulfate salt (~300 MIC); tobramycin and colistin low (tobramycin + colistin low): 100 µg/mL tobramycin and 80 µg/mL colistin sulfate salt; 1000 µg/mL tobramycin (1000 MIC). For the light assisted antibiotic treatment; 100 µg/mL tobramycin were delivered after UVB was applied (sunlight equivalent, 20.000 J/m^2^). The antibiotics were added into the ABTG agar plates and biofilms were treated by moving the biofilm growing on nitrocellulose filters to the antibiotic containing media plates.

### CFU determination

The method for quantitative bacteriology is described in Argyraki *et al*.^6^; and distinct samples were used for the CFU determination. Following treatment the filter biofilm was transferred with sterile forceps to 5 mL saline (0.9% NaCl) and detached through sonication in an Branson B2510-DTH ultrasonic cleaner (5 min degas followed by 5 min sonication). Serial dilutions in the cases of UVB irradiation treatment versus topically administrated antibiotics and light assisted antibiotics were performed from 10^0^ (no dilution) to 10^−7^ and the spotted volume was 10 μL performed in triplicate; resulting in a detection limit of 100 bacteria per ml.

### Statistics

All treatments were performed on three different biological replicates (n = 3), based on two or three technical replicates, as a standard for testing reproducibility. The statistical dispersion was measured as standard deviation, reported in errors, and is of biological origin if not stated otherwise in the text. One-way ANOVA or two-way ANOVA followed by Bonferoni corrected multiple comparison were performed in GraphPad Prism 7.01.

## Data Availability

The datasets generated during and/or analyzed during the current study are available from the corresponding author on reasonable request.
